# Novel microfilariae detected in Galápagos passerines

**DOI:** 10.1016/j.ijppaw.2025.101115

**Published:** 2025-07-12

**Authors:** Diana Carolina Loyola, Allyson Placko, Birgit Fessl, Sabrina M. McNew

**Affiliations:** a Department of Ecology & Evolutionary Biology, University of Arizona, Tucson, AZ, 85719, USA; b Charles Darwin Research Station, Charles Darwin Foundation, Santa Cruz, Galápagos Islands, Ecuador

**Keywords:** Microfilaria, Eufilaria, Galápagos islands, COI barcoding, Nematode

## Abstract

Emerging parasites pose a serious risk to the health and survival of wild animal populations, particularly on islands where species often lack prior exposure and evolved defenses. We present the first report of a novel microfilaria infection found in blood from six species of Galápagos passerines in the coastal zone of Santa Cruz Island. Across 13 months, spanning two wet seasons and one dry season, 294 individuals were sampled and evaluated for microfilarial presence through microscopy and/or polymerase chain reaction. We barcoded the mitochondrial Cytochrome *c* oxidase I (COI) gene to tentatively place this microfilaria in the genus *Eufilaria*. We found host species level variation in infection, with certain species, like the vegetarian finch (*Platyspiza crassirostris*) and the common cactus finch (*Geospiza. scandens*) having very high prevalence, while others, like the Galápagos mockingbird (*Mimus parvulus*) and small tree finch (*Camarhynchus parvulus*) showing significantly lower prevalence. We investigated leukocyte counts, H/L ratios and body condition to evaluate the potential effects of infection on birds and found no relationship between infection status and these health indices. We also tested to see if seasonality could predict the infection trends found in our data and found a relationship with monthly rainfall, where more rain predicts higher microfilarial prevalence. Although we cannot confirm exactly when this parasite established in the Galápagos, our study highlights the importance of continued disease surveillance in endemic systems and underscores the need for species-level COI barcodes to improve microfilaria identification and phylogenetics.

## Highlights

• Microfilariae reported for the first time in the blood of Galapagos passerines
• Barcoding of the COI gene places the parasites in the genus *Eufilaria*
• Phylogenetic analysis shows this parasite is separate from seabird microfilaria
• Prevalence varies among host species and is correlated with rainfall

## Graphical abstract

**Figure:**
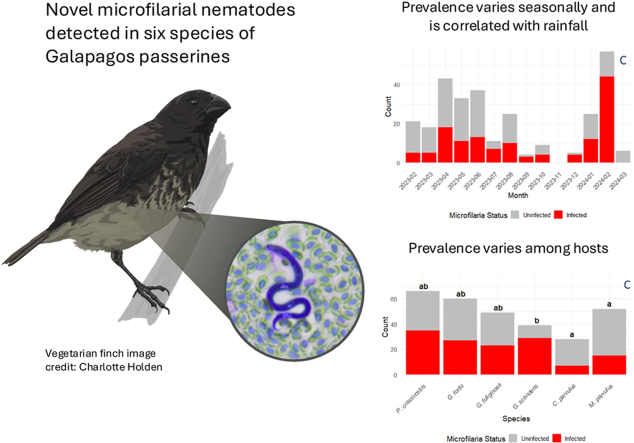

## 1 Introduction

The spread of parasitic diseases into novel host populations poses a significant threat to biodiversity. The risk for isolated populations, such as species endemic to islands, is particularly high, as immunologically naive hosts may be unable to mount an effective response to infection (Addesso et al., 2020; Wyatt et al., 2008). For example, the arrival of European settlers to the Hawaiian archipelago brought infectious diseases such as avian malaria (*Plasmodium relictum*) and avian pox (*Avipoxvirus*), which subsequently drove population declines and extinction of several species of honeycreepers (Carduelinae) (van Riper III et al., 2002; Williams et al., 2021). Understanding the factors that drive the emergence of novel parasites in host populations and their subsequent effects on those hosts is crucial for conservation.

The Galápagos Islands are a volcanic archipelago located roughly 1000 km off the coast of mainland Ecuador, renowned for their unique and endemic wildlife. The endemic birds of the Galápagos include adaptive radiations of mockingbirds and finches, made famous for their role in shaping Darwin's ideas about the transmutation of species (Sulloway, 1982). Evolving in isolation, they have adapted to endure strong seasonal shifts in resource availability between the dry, cool season (June to December) and the warm, wet season (January to May) (Grant and Grant, 2014; Trueman and d’Ozouville, 2010). However, climate change is increasing variability in these seasonal patterns, leading to more frequent and unpredictable El Niño events, which bring excess rainfall and temporarily boost food resources, as well as La Niña cycles, which cause unusually long periods of drought (Grant and Grant, 2014). Climatic shifts may have important effects on the presence of parasites and pathogens as well. Increases in temperature and rainfall support the growth of mosquito and other arthropod vector populations, potentially driving increased transmission of vector-borne parasites (Lynton-Jenkins et al., 2021; Parker et al., 2011).

While this isolated evolution has allowed Galápagos endemic life to thrive on the islands, it can also have serious consequences for species’ survival. Introduced parasites represent some of the biggest threats to Galápagos bird populations. The avian vampire fly (*Philornis downsi*) is an introduced nest parasite that causes significant mortality in nestling passerines, in some populations reducing reproductive success to nearly zero (Fessl et al., 2001; McNew and Clayton, 2018). Another example is avian pox, which has become highly prevalent in Galápagos bird populations over the past century (Parker et al., 2011). Monitoring these endemic bird populations for emerging diseases is important for their conservation and management.

Microfilarial nematodes are an often-overlooked parasite belonging to the superfamily Filarioidea. They can be found in several orders of wild birds across the world, particularly in areas with elevated temperatures and humidity (Moens et al., 2016; Ricklefs et al., 2016). Adult filaroid worms reside in the body cavity or specific tissues, such as the brain. The life cycle begins with reproduction and the release of larval stages (“microfilariae”) into the bloodstream. The larval nematodes then are transmitted to a new host through a hematophagous vector, like mosquitos, biting midges or mites. The microfilariae develop in the vector into the L3 or infective larvae before entering a new host (Bartlett, 2008).

Identification and study of microfilarial nematodes is difficult because of their life cycle. Adult filariid worms inhabit tissues or the body cavity and are difficult to sample from living hosts (Bartlett, 2008; Binkienė et al., 2021). Instead, detection most often occurs incidentally through sampling of larvae in the peripheral blood, which cannot be identified to species morphologically. Furthermore, taxonomic identification through molecular barcoding is difficult due to a lack of genetic information for this group (Bartlett, 2008). In addition to the challenges of identification, the ecology of microfilarial nematodes is obscure. The environmental and host factors that govern the prevalence and distribution of these parasites are poorly known. The activity of the larval stages of many species is highest in the evening or night, presumably when vector activity and the probability of transmission is also highest (Anderson, 2000; Vaughan et al., 2021). As a result, prevalence may vary or be underestimated if hosts are primarily sampled at other times of the day. Little is known about the effects of microfilarial nematodes on host health. In some cases, infections can lead to inflammation of blood vessels, or cellular stress, adversely affecting host fitness (Bartlett, 2008; Binkienė et al., 2021). In other studies, however, filarial nematodes have minimal impact on host health (Bartlett, 2008; Santiago-Alarcon and Merkel, 2018).

The earliest recorded observations of helminths in Galápagos birds dates to 1868, where Dr. Simeon Habel reported “parasitic worms” under the skin of the head of the common cactus finch (*Cactornis assimilis,* now recognized as *Geospiza scandens*) as well as short eared owl (*Asio flammeus galapagoensis*) (Salvin, 1876). These observations suggest a longstanding presence of helminths in landbirds in the region; however, these parasites have not been identified morphologically or genetically. Microfilaria have more recently been detected in Galápagos penguins (*Spheniscus mendiculus*) and flightless cormorants (*Phalacrocorax harrisi*) on Isabela Island (Merkel et al., 2007). Over the past several decades, the Galápagos Islands have experienced a dramatic increase in anthropogenic disturbance, driven by the rise in tourism and development on the islands (Toral-Granda et al., 2017). Along with notable parasitic arthropods such as *P. downsi*, populations of other introduced dipteran parasites have increased on the islands, raising the possibility that new vector-borne parasites will emerge in endemic Galápagos host populations.

Here, for the first time, we document microfilarial nematode (hereafter “microfilaria”) infections in blood samples collected from five species of Darwin's finch as well as the Galápagos mockingbird (*Mimus parvulus*) living in the arid coastal environment of the Galápagos. We document the prevalence of this microfilaria over 13 months using two methods: microscopy and molecular screening. We then investigate whether the microfilaria found in the passerines is different from the species previously found in seabirds on Isabela Island. Finally, we test whether the prevalence of infection is associated with climatic variation or particular host species, and whether infection is correlated with host condition or immune response.

## 2 Materials and methods

### 2.1 Study area and sample collection

This study was conducted from February 2023 to March 2024. The field work was conducted at two sites on the southern coast of Santa Cruz Island, “El Barranco” (−0.7386212, −90.3017273) near the Charles Darwin Research Station on the outskirts of Puerto Ayora, and “Garrapatero” (−0.686262°, −90.222413°). All procedures were approved by the University of Arizona IACUC (no. 2022-1000), and Galápagos National Park. Birds were primarily sampled at the Barranco site; a small number of samples (n = 12) were taken at the Garrapatero site. We used two to four mist-nets (9 × 3 m and 12x3 and 36 mm mesh size) to capture birds from 6:00 h to 9:30 h per day. Birds were identified to species, measured and banded with a uniquely numbered aluminum leg band. Approximately 70 μL of blood was collected from the brachial vein using sterile needles and a heparinized capillary tube. We used a drop of blood to prepare blood smears on a slide, and fixed in methanol for 2 min in the field. The remainder of the blood sample was preserved in Longmire's buffer [100 mM Tris pH 8; 100 mM Na 2 EDTA, 10 mM NaCl, 2.0 % SDS] (Hoelzel, 1992). We focused on the Galápagos mockingbird *(Mimus parvulus*) and five common finch species: the vegetarian finch (*Platyspiza crassirostris*), medium ground finch *(Geospiza fortis*), small ground finch (*G. fuliginosa*), common cactus finch (*G. scandens*) and the small tree finch*, (Camarhynchus parvulus*)

### 2.2 Microscopy detection and leukocyte profiling

We stained slides using the JorVet Dip Quick Stain solution kit (Jorgensen Laboratories, Loveland, CO) following manufacturer's protocol. One author (CDL) inspected slides using a Euromex compound microscope at 100× magnification under oil immersion. First, 25 fields were scanned and the number of erythrocytes was quantified using the CFU.ai app v1.4. Then, white blood cells (lymphocytes, heterophils, monocytes, eosinophils, and thrombocytes) were quantified in either 100 fields or 50 leukocytes, whichever was reached first. For statistical analysis, these raw leukocyte counts were scaled by erythrocyte number/1000 to correct for screening effort. The counts can inform the overall health of the birds, through the visualization of blood parasites, red blood cells and white blood cells. The presence and abundance of different types of white blood cells provide a snapshot of the immune system which can be used to inform a bird's immune strategy to infection (Davis et al., 2008). In addition to leukocyte counts, we used the heterophil to lymphocyte (H/L) ratio to assess stress and health (Skwarska, 2019). During slide screening, the presence or absence of any microfilaria was recorded, and if present, a representative parasite was photographed. A total of 156 samples from individual birds were screened by microscopy.

### 2.3 Molecular barcoding

A subset of total samples taken were exported to the University of Arizona for PCR barcoding. Total DNA was extracted using Qiagen Blood and Tissue Kit (Hilden, Germany, Catalogue # 69504) following the manufacturer's instructions with slight modifications, namely increasing incubation time to 2–3 h. PCR protocols followed methods described in Merkel et al. (2007). We amplified a 688 bp fragment of mitochondrial cytochrome *c* oxidase subunit 1 gene using the COIinf F/R primers designed for the family Onchocercidae (Filarioidea) (COIintF 5′-TGATTGGTGGTTTTGGTAA-3′ and COIintR 5′-ATAAGTACGAGTATCAATATC-3′) (Casiraghi et al., 2001). A PCR master mix was prepared with 12.5 μL of Green Hot start DreamTaq, 1 μL of 10 mM forward and reverse primers, 8 μL of water and 2.5 μL of template DNA for a total reaction mix of 25 μL. A touch-down PCR was conducted with cycling conditions as follows: initial denaturation for 2 min at 94 °C; followed by 8 cycles of a touchdown cycle starting with denaturation at 94 °C for 45 s, annealing starting at 51 °C and decreasing every cycle by 0.5 °C and then extension for 90 s at 72 °C; followed by another 25 cycles with denaturation at 94 °C for 45 s, annealing at 45 °C for 45 s, and extension at 72 °C for 90 s; followed by a final extension of 72 °C for 7 min. Samples were run for 45 min on 1.5 % agarose gel to determine samples positive for infection. A total of 147 individuals were screened via PCR. Nine individuals were evaluated by both microscopy and molecular methods. A subset of positive samples (n = 30) with appropriately sized amplicons spanning all species were prepared for sequencing using a Qiagen QIAquick PCR cleanup kit (Hilden, Germany, Catalogue #28104), following manufacturer instructions. Samples were sequenced via Sanger sequencing at the University of Arizona Genetics Core.

### 2.4 Phylogenetic analysis

We manually inspected sequences and trimmed or corrected errors based on chromatograms. We obtained sequences to use in phylogenetic analysis from GenBank after identifying sequences of interest from BLAST or other reconstructions of Onchocercidae phylogeny (Table 1). We aligned our sequences with these other published sequences of interest and trimmed to produce a 530 bp alignment with no gaps using Geneious (v2024.0.7).

**Table 1:** Sequences used in generation of Onchocercidae phylogeny.

Parasite species	Host class	Host species	GenBank ID	Locality
*Eufilaria acrocephalusi*	Bird	*Acrocephalus scirpaceus*	MT800769	Lithuania
*Filarioidea* sp. DBB-2016-III	Unknown	*Culex pipiens∗*	LC107819	Spain
*Filaria* sp. 150413SPB16	Bird	*Quiscalus mexicanus*	MH379968	USA: Texas
*Eufilaria sylviae*	Bird	*Sylvia borin*	MT800770	Lithuania
*Onchocercidae* sp. MI1 GLH-2012	Bird	*Quiscalus quiscula*	JQ867058	USA: Chicago
*Splendidofilaria* sp. GLH-2012	Bird	*Cyanocitta cristata*	JQ867061	USA: Chicago
*Aproctella* sp. MIB ZPL 02719	Bird	*Turdus rufiventris, Saltator similis*	FR823331	Brazil
*Chandlerella quiscali*	Bird	*Quiscalus quiscula*	JQ867067	USA: Chicago
*Mansonella ozzardi*	Mammal	*Homo sapiens*	KP760195	Haiti
*Mansonella dunni*	Mammal	*Tupaia glis*	KY434309	Malaysia
*Loa loa*	Mammal	*Homo sapiens*	AJ544875	Cameroon
*Dipetalonema gracile*	Mammal	*Cebus olivaceus*	AJ544877	Venezuela
*Acanthocheilonema viteae*	Mammal	Rodent spp.	AJ272117	USA
*Thelazia callipaeda*	Mammal	*Canis lupis familiaris*	AM042555	China
*Onchocerca ochengi*	Mammal	*Bos taurus* (cattle) ∗∗	AJ271618	Cameroon
*Onchocerca lupi*	Mammal	*Canis lupis familiaris*	AJ415417	Hungary
*Onchocerca gutturosa*	Mammal	*Bos taurus* (cattle) ∗∗	AJ271617	Cameroon
*Onchocerca gibsoni*	Mammal	*Bos taurus* (cattle)∗∗	AJ271616	Australia
*Dirofilaria repens*	Mammal	*Canis lupis familiaris*	AJ271614	Italy
*Dirofilaria immitis*	Mammal	*Canis lupis familiaris*	AJ271613	Italy
*Splendidofilaria mavis* isolate 962_F	Bird	*Turdus merula*	OK631737	Lithuania
*Splendidofilaria bartletti* isolate 901A_MF	Bird	*Sylvia atricapilla*	MT800764	Lithuania
Nematoda sp. NKW-2006 isolate 70	Bird	*Spheniscus mendiculus*	DQ838574	Ecuador: Galapagos Islands
Nematoda sp. NKW-2006 isolate 52	Bird	*Phalacrocorax harrisi*	DQ838573	Ecuador: Galapagos Islands
Nematoda sp. NKW-2006 isolate 38	Bird	*Phalacrocorax harrisi*	DQ838572	Ecuador: Galapagos Islands
Nematoda sp. NKW-2006 isolate 65	Bird	*Spheniscus mendiculus*	DQ838571	Ecuador: Galapagos Islands
Nematoda sp. NKW-2006 isolate 1	Bird	*Phalacrocorax harrisi*	DQ838570	Ecuador: Galapagos Islands
*Onchocercidae* sp. 10MF3 GLH-2012	Bird	*Turdus migratorius*	JQ867065	USA: Chicago
Galapagos Passerine Haplotype I (this study)	Bird	Various	Table S1	Ecuador: Galapagos Islands
Galapagos Passerine Haplotype II (this study)	Bird	Various	Table S1	Ecuador: Galapagos Islands

∗Parasite was identified from vector, vertebrate host unknown.

∗∗Host species given as "cattle," scientific name inferred.

We used the IQ-TREE web server's model selection to determine the best-fit phylogenetic model: the General Time Reversible (GTR) model with empirical base frequencies (+F), a proportion of invariable sites estimated from the data (+I), and gamma-distributed rate heterogeneity across sites modeled with four discrete rate categories (+G4), we then used the program BEAST (v2.7.7 2024) to estimate the phylogenetic relationships among microfilariae with an MCMC chain length of 1,000,000 and a 10 % burn in. The final tree was then visualized using Figtree (v1.4.4).

### 2.5 Ecological analyses

We tested for ecological and host correlates of microfilaria infection using generalized linear models. All analyses were performed with R (v4.4.3) in R Studio (v2024.09.0). Because we used two different methods of detecting infection (microscopy and PCR), all analyses were run separately on the two datasets as well as on the combined dataset, unless otherwise noted. Only nine samples were tested by both microscopy and PCR; for those nine samples only the PCR result was used in the combined dataset.

The prevalence of vector-borne parasites is often correlated with rainfall, as many dipteran vectors require water to complete their larval stage (Gage et al., 2008). If prevalence is driven by rainfall, we would reasonably expect some time lag between precipitation, vector development, and subsequent appearance in the vertebrate host. However, because we lack information on the life history of the microfilaria and its vector, we did not parameterize how long that lag would be. Instead, we simply tested for a correlation between microfilaria prevalence in any particular month and the total rainfall that fell in that month. We accessed daily rainfall data from the Charles Darwin Foundation's Climatology Database (CDF Climatology Database, 2025). We calculated total cumulative rainfall per month as a measure of climatic conditions over the study period. To test for a relationship between microfilaria infection rates and total rainfall we created a generalized linear model with binomial distribution. The response variable was a paired vector of the number of infected and uninfected individuals for the month and the predictor variable was total precipitation. Next, we investigated whether there was significant variation in microfilaria prevalence among host species using a generalized linear model with binomial distribution with microfilarial infection as the binary response variable and species as the predictor variable. Significant differences between species were assessed using Tukey post-hoc tests in the *emmeans* package. Then, we tested whether microfilaria infection was associated with reduced body condition or elevated leukocyte counts. We calculated body condition as a scaled mass index (SMI) using individual mass and tarsus length (Peig and Green, 2009). We modeled body condition using the combined data set (results from microscopy and PCR) and white blood cell counts from the microscopy data set as response variables in linear mixed effects models, with fixed predictor variables of microfilaria infection and sex, and random predictor of species. We ran separate models for each leukocyte type. We corrected p values of leukocyte models for multiple hypothesis testing using the p.adjust function with the “Holm” method in R.

## 3 Results

In total, 136 out of 294 birds from six species tested positive for microfilaria infection (Table 2). Of the 147 birds tested via PCR, 95 (64.6 %) were positive. Prevalence was lower using microscopy; of the 156 birds tested, 46 (29.5 %) had visible microfilaria in slide samples. Of the nine samples tested with both microscopy and PCR, three tested negative with both methods, five tested positive with PCR, but negative with microscopy. One sample tested positive for microfilaria from microscopy but tested negative with PCR. Prevalence varied among species and was highest in *P. crassirostris* and *G. scandens* (Table 2)

**Table 2:** Prevalence of microfilaria infection per species by PCR and microscopy.

Species	Birds infected/tested using PCR	Birds infected/tested using microscopy
Vegetarian finch *Platyspiza crassirostris*	21/23 (91 %)	14/43 (33 %)
Medium ground finch *Geospiza fortis*	17/26 (65 %)	10/34 (29 %)
Small ground finch *Geospiza fuliginosa*	19/28 (68 %)	5/22 (22 %)
Common cactus finch *Geospiza scandens*	21/28 (75 %)	10/15 (66 %)
Small tree finch *Camarhynchus parvulus*	7/16 (44 %)	0/13 (0 %)
Galápagos mockingbird *Mimus parvulus*	10/26 (38 %)	7/29 (24 %)
*Total*	95/147 (64.6 %)	46/156 (29.5 %)

### 3.1 Morphology

**Fig. 1:**
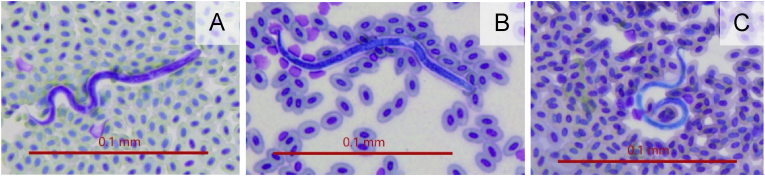
Examples of microfilarial nematodes visible on blood smears from different host species. A: common cactus finch *(G. scandens*); B: vegetarian finch *(P. crassirostris*); C: Galápagos mockingbird (*M. parvulus*).

All microfilaria had similar morphology, regardless of host species (Fig. 1). The larval parasites found in the peripheral blood are elongated and thread-like, with a slightly tapered, rounded head, slender pointed, slightly curved tail and appear to be unsheathed. The average length was 127.5 μm (standard deviation = 0.0141, N = 12) and the average width 5.5 μm (standard deviation = 0.0005, N = 12).

### 3.2 Phylogenetics

**Fig. 2:**
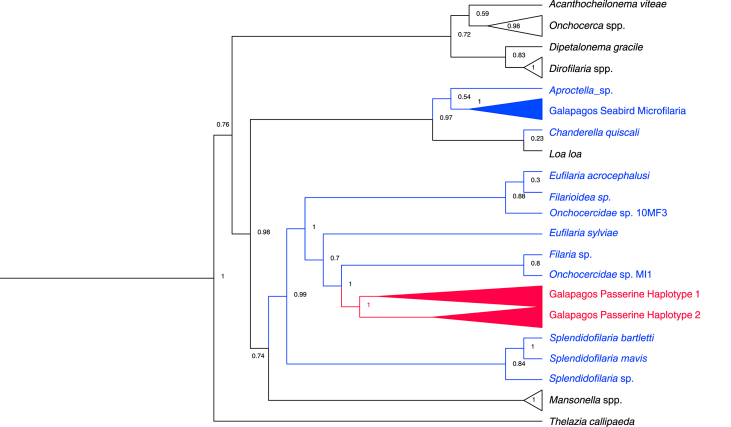
Phylogenetic reconstruction of genetic relationship of microfilaria found in Galápagos passerines, which are colored in red. Other microfilaria found in birds are colored in blue while those infecting mammals are in black. The other microfilaria found in Galápagos seabirds is from a different clade than the microfilaria sequenced in this study.

We sequenced 30 positive samples and found two haplotypes differentiated by three base pair substitutions within the 540 bp COI fragment. We found that 20 samples matched “haplotype 1” while the remaining 10 were “haplotype 2”. Haplotype 1 was recovered from all six host species, while haplotype 2 was found in five species (all except *G. fortis*). The sequences obtained most closely matched other undetermined species, but they fall within a clade containing two named species from the genus *Eufilaria* Seurat, 1921 with high branch support. We therefore tentatively identify the microfilaria infecting Galápagos passerines as species within the *Eufilaria* genus. The microfilaria infecting Galápagos seabirds forms a distinct clade from the sequences generated in this study, supporting the inference that the microfilariae infecting seabirds and passerines in the Galápagos represent different species, likely belonging to separate genera (Fig. 2).

### 3.3 Ecology

#### 3.3.1 Climate

**Fig. 3:**
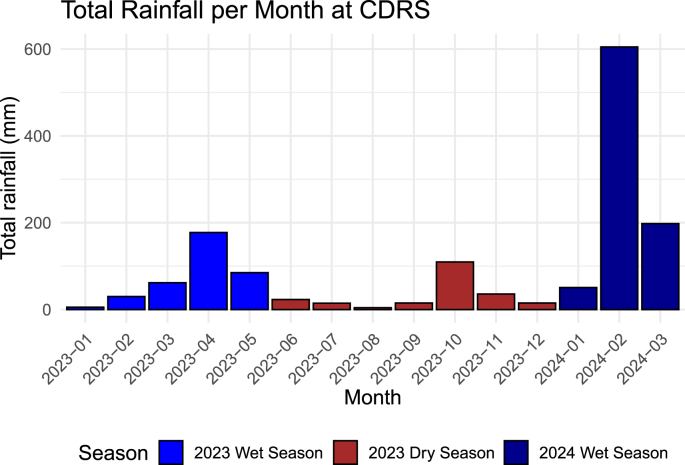
Monthly rainfall over the study period recorded at the Charles Darwin Research Station. Colors represent the typical dry and wet seasons in the Galápagos Islands (Grant and Grant, 2014; Trueman and d’Ozouville, 2010); however, it is important to note that rainfall patterns in the region are highly variable.

**Fig. 4:**
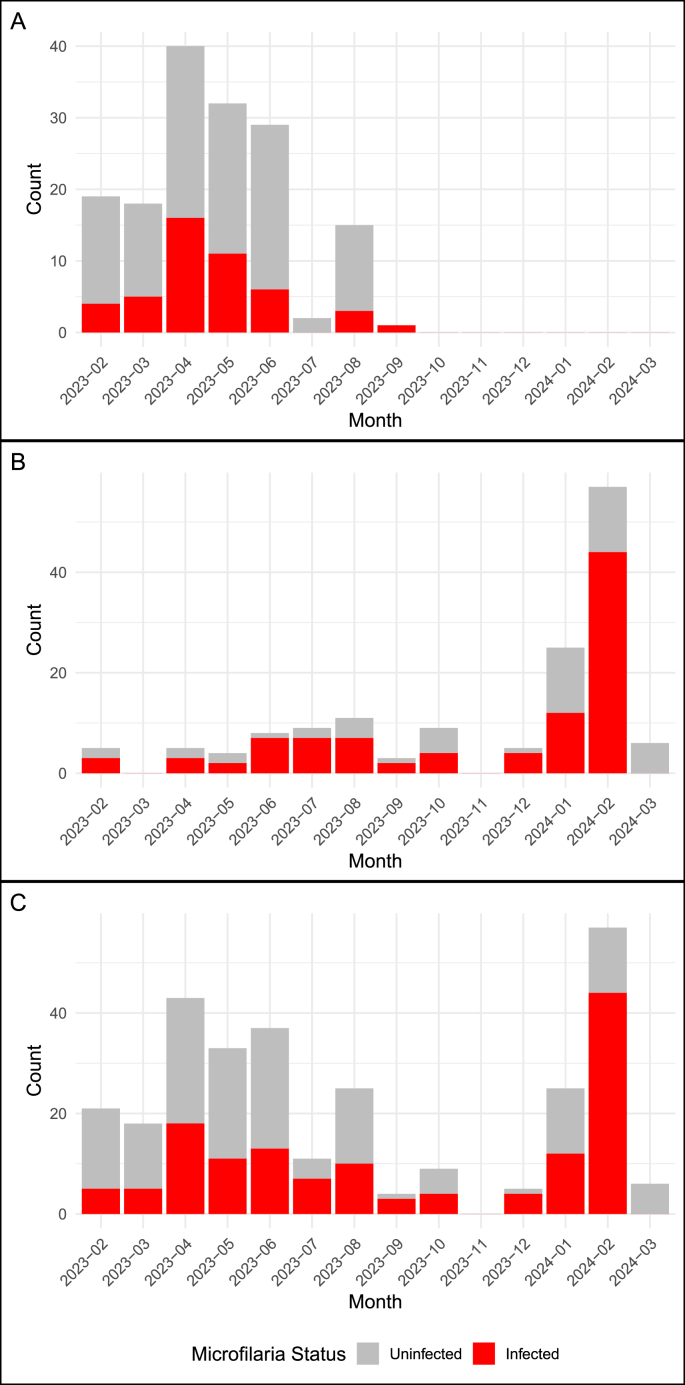
Counts of positive microfilaria for each month of the study period. A) the microscopy data set, B) the PCR data set and, C) the combined data set. All three panels are shown on the same temporal scale for comparability; note that the sampling period of the microscopy and PCR datasets do not completely overlap.

Since microfilariae are transmitted through hematophagous vectors, we predicted that seasonal variation in rainfall would be an important driver of microfilarial prevalence. We tested for a correlation between total monthly rainfall and microfilaria prevalence using both methods of detection as well as a combined data set (Fig. 3, Fig. 4). Rainfall was significantly positively associated with infection using PCR (β [log odds] = 0.0013, 95 % CI = 4.03∗10^−5^ – 0.003; p = 0.047) and microscopy datasets (β [log odds] = 0.0056, 95 % CI = 2.61∗10^−4^– 0.011; p = 0.04) as well as when both datasets were combined (β [log odds] = 0.0028, 95 % CI = 0.002–0.004; p < 0.001).

#### 3.3.2 Host

**Fig. 5:**
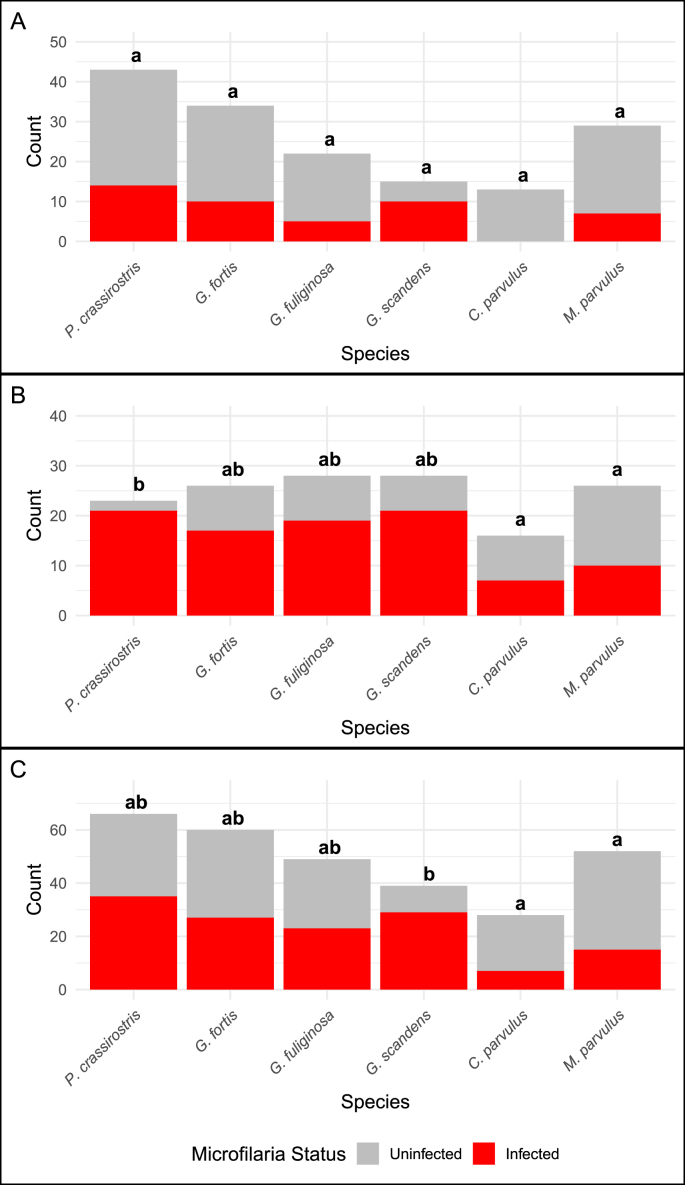
Variation in microfilaria prevalence across species and data set. Letters above bars indicate results of Tukey post hoc tests evaluating differences in prevalence among species. Species marked with different letters have significant differences in microfilaria prevalence. A) Results from microscopy. Species did not vary significantly in prevalence. B) Results from PCR showing significantly higher prevalence in *P. crassirostris* (vegetarian finch) compared to *M. parvulus* (Galápagos mockingbird) and *C. parvulus* (small tree finch). C) Results from combined dataset. Again, *M. parvulus* and *C. parvulus* have the lowest prevalence.

We did not detect any significant differences among species in microfilaria prevalence using microscopy (p > 0.05) (Fig. 5). However, some species level variation was found through PCR and the combined data (Fig. 5). Using infections detected through PCR, *P. crassirostris* had significantly higher prevalence (91 %) than *M. parvulus* (38 %) (adjusted p = 0.01) and *C. parvulus* (44 %) (adjusted p = 0.04)*.* No other significant differences were found between species (p > 0.05). Using a combined dataset of both PCR and microscopy detections, *G. scandens* has the highest rates of infection, and its prevalence is significantly higher than *M. parvulus* (adjusted p = 0.002) and *C. parvulus* (adjusted p = 0.0005). We found no differences in prevalence between male or female hosts in any dataset (p > 0.05 for all analyses). Nearly all birds sampled in our study were adults; however, we did include samples from eight nestlings (two *G. scandens* and six *M. parvulus*), all of which tested negative for infection.

**Fig. 6:**
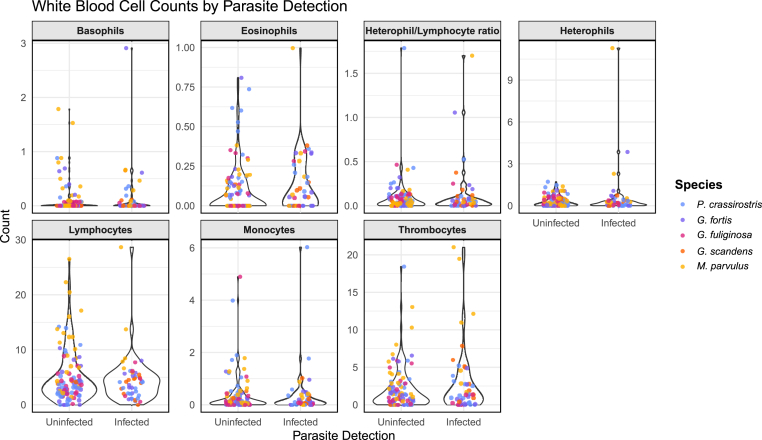
Comparison of counts per 1000 erythrocytes of different white blood cell types as well as heterophil/lymphocyte ratio between microfilaria infected and uninfected individuals. Each colored point represents an individual, colored by species. No significant differences are detected for any of the cell types between infected and uninfected individuals. *C. parvulus* was excluded from this analysis as no individual was positive for infection using microscopy.

**Fig. 7:**
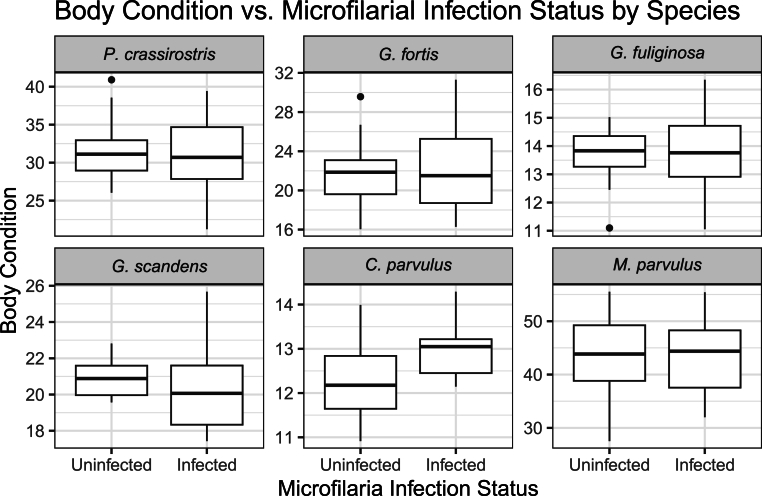
Comparison of body condition (scaled mass index) between infected and uninfected birds for each of the six species included in the study. No significant differences were detected between infected and uninfected birds for any of the species.

We investigated the impact microfilarial infection had on the bird's health through the proxy of body condition, H/L ratios, and white blood cell counts. Infection status had no significant effect on any leukocyte count or H/L ratios (p > 0.05 for all analyses; Fig. 6). Likewise, infection status had no significant effect on body condition (p > 0.05 for all analyses; Fig. 7).

## 4 Discussion

### 4.1 Identification and origin

We report microfilarial infections in six species of endemic passerines on Santa Cruz Island, Galápagos. Although the anecdotal reports of “parasitic worms” in Galápagos landbirds date to the 19th century, this is the first time that genetic and morphological data confirm the presence of filarial nematodes in endemic Galápagos passerines. Based on genetic barcoding, we are confident this species is different from the microfilaria found in infected sea birds on Isabela Island; however, we are unable to identify the parasites in this study to species without further morphological study. Our results provide additional new data on the ecology and health effects of this parasite, suggesting that its prevalence is associated with rainfall, that it is common in several host species, but that it does not have strong effects on host health or immune parameters.

We found variation in estimates of prevalence using our two screening methods, with infections more frequently detected using PCR compared to microscopy. Due to sampling and export constraints, samples were tested either by microscopy or PCR, but rarely both. As a result, we did not directly compare the sensitivity and specificity of screening methods on the same set of samples. In rare cases we do expect PCR screening to produce false negatives, particularly when infection intensity is low and/or DNA extraction concentration is low. Nevertheless, our results suggest that microscopy more frequently underestimates infection prevalence, likely due to the small amount of blood screened as well as microfilarial periodicity. Studies of other endoparasites including avian malaria have similarly shown that PCR is a more sensitive detection method, capable of detecting low intensity infections better than microscopy (Llewellyn et al., 2016; Reinoso-Pérez et al., 2024; Valkiūnas et al., 2008). We acknowledge that PCR has the potential to result in false positives, as a positive PCR result could be obtained from a microfilarial parasite that has already been killed by the immune system or potentially non-specific amplification. While both data sets are valuable for different applications, we suggest that PCR is a more accurate representation of overall prevalence and should be the main method by which infection should be assessed for non-destructive field sampling. Regardless of method, prevalence in this system is remarkably high, given that other studies rarely report microfilaria prevalence above 15 % in wild birds (Garrido-Bautista et al., 2023; Golnar et al., 2021; Hamer et al., 2013; Sehgal et al., 2005). Previous studies have found that island populations can be hotspots of microfilaria infection; our results similarly indicate that island birds may be particularly susceptible to parasitic nematodes (Garrido-Bautista et al., 2023; Sehgal et al., 2005).

We found two distinct haplotypes among the parasites we sequenced, suggesting that there is some genetic variation in our microfilaria population. However, given the small differences between the two haplotypes (<1 % divergence between haplotypes) and the fact that the haplotypes did not segregate among host species, we suggest that the microfilaria we found in Galápagos passerines on Santa Cruz belong to one single species. Notably, the microfilaria we detected form a monophyletic clade that is not sister to the microfilaria previously found in Galápagos seabirds on Isabela and Fernandina (Merkel et al., 2007). Thus, our data suggest that distinct microfilaria species infect land and seabirds in the Galápagos. Comparison of our sequences with species identified published COI sequences suggests that our microfilaria belongs to the genus *Eufilaria*. The closest relatives to the parasite in our study include similarly unspecified nematodes infecting passerines in the United States. Filaria_sp.*150413SPB16 (*MH379969*)* that was detected in great-tailed grackles (*Quiscalus mexicanus*) in Texas and an Onchocercidae sp. MI1 GLH-2012 (JQ867058), which was discovered in American robins (*Turdus migratorius*) and house sparrows (*Passer domesticus*) in Illinois (Golnar et al., 2021; Hamer et al., 2013). The next closest relative is a named species, *Eufilaria sylvia* Binkienė et al., 2021*,* which was discovered in another passerine species, the garden warbler (*Sylvia borin*) in Lithuania (Binkienė et al., 2021). Both named species of Eufilaria with COI sequences, *E. sylvia* and *E. acrocephalusi* Binkienė et al., 2021, were included in our tree. Although neither clustered particularly closely with our sequences, they comprised a monophyletic clade with our sequences and several other sequences missing a genus or species determination. Additionally, the morphology of our microfilaria is similar to other *Eufilaria* microfilariae descriptions (Anderson, 2000). Thus, while description of our parasite species will require additional morphological and phylogenetic study, we think it likely that this microfilaria is a novel species within the *Eufilaria* genus.

Investigation of the biology of this parasite may provide insight into its origin in the Galápagos. *Eufilaria* is a genus of the microfilariae-producing family, Onchocercidae, and has been primarily detected in passerine birds (Anderson, 2000). Adults are typically localized around the head and neck of the host but have also been found in subcutaneous tissue around the larynx, esophagus, and crop, as well as in the subcutaneous tissue of the leg joints (Anderson, 2000; Bartlett, 2008). *Eufilaria* spp. are vectored by ornithophilic ceratopogonid flies (Anderson, 2000). Eleven species from the family Ceratopogonidae are recorded in the Galápagos Islands, including endemic species, such as *Forcipomyia galapagensis* Wirth 1956 and *Dasyhelea spathicercus* Wirth 1969, as well as introduced species, such as *Culicoides pusillus* Lutz 1913; Borkent (1991)*.* One unknown species in the genus *Forcipomyia* is known from just one collected female. Its morphological description as well as its tentative placement in the subgenus *Lepidohelea* indicate that it is likely a blood feeder (Borkent, 1991). However, since this species is defined by a single individual collected in the 1990s and has not been recorded since, we think this species is unlikely to be the vector of the parasites in our study (Borkent, 1991; Sinclair, 2023). Seven of the remaining ten ceratopogonids found in Galápagos belong to the genus *Dasyhelea* Kieffer, 1911 which exclusively feed on nectar and thus are unlikely to be vectors (Dominiak and Borkent, 2023). Of the remaining three, *F. galapagensis* has mouthparts that are described as similar to others which feed on caterpillars, suggesting this species is unlikely to feed on vertebrates (Borkent, 1991). Finally, *C. pusillus* and *F. genualis* Loew 1866 are known parasites of vertebrates, implicating these two species as possible vectors of the *Eufilaria* sp. in our study. Both species feed on mammals and while only *C. pusillus* has been recorded biting birds, the lack of research into *F. genualis* suggests they should not be excluded as a potential vector (Borkent, 1991).

Both putative vectors, *C. pusillus* and *F. genualis* were first recoded in 1964 and are believed to be introduced to the Galápagos through human activity due to their association with agriculture (Borkent, 1991; “CDF Galápagos Species Database,” 2025). Although we cannot exclude the possibility of other potential vectors for our *Eufilaria* sp. parasite, the fact that the most likely vectors are introduced species suggests that the parasite we detected could also be introduced to the islands. The mid-20th century arrival of the proposed vectors of the *Eufilaria* sp. in our study is difficult to reconcile with reports of parasitic worms in *G. scandens* nearly a century earlier (Salvin, 1876). It is possible that the vectors and associated parasites arrived in the archipelago earlier than thought, and/or that the parasites that Habel observed were unrelated to the ones we found. Future work is needed to definitively identify the vector of the parasites in our study. However, determining exactly when and how it arrived in the Galápagos will be challenging without the ability to genotype parasites from historical samples.

### 4.2 Parasite ecology and effects on hosts

We hypothesized that microfilariae infection rates would be highest during the rainy months, as many ceratopogonid species reproduce in water or in rotting plant materials (Sinclair, 2023). We found that rainfall was positively associated with microfilaria prevalence, indicating that climatic conditions have an important effect on the parasite's transmission and/or reproductive success. The ecology of the Charles Darwin Research Station, where most of our samples were collected, could have also influenced infection rates. The station's proximity to the main population center in the Galápagos and more consistent presence of water may attract higher densities of vectors and susceptible hosts. However, a small subset of birds from another coastal, more arid site, Garrapatero, also tested positive for microfilaria (N = 4/12 samples), suggesting that this parasite is not limited to urban areas. Future work is needed to investigate the distribution and habitat breadth of this parasite across the archipelago.

We investigated host characteristics that could be associated with differential susceptibility to infection. We lacked the data to statistically test for variation in infection rates with host age. However, we observed that eight nestlings in our analysis tested negative via PCR. Biting midges are described as weak fliers which could explain the lack of nestling parasitism despite nests often being attractive to flying vectors due to the increased CO2 (Castaño-Vázquez et al., 2020). The most notable predictor of variation in prevalence was host species. *Platyspiza crassirostris* had very high prevalence of filarial infection, with 91 % of screened birds testing positive for infection through PCR while other species, like the *M. parvulus* and *C. parvulus* have much lower rates of infection, 36 % and 44 % testing positive respectively. Not all post-hoc comparisons revealed significant differences between other species with intermediate prevalences (Fig. 5). These PCR results contrast with the microscopy and combined data sets, with microscopy finding no significant differences between the species and the combined having *G. scandens* as significantly different from *M. parvulus* and *C. parvulus*. Nevertheless, this variation suggests that differences in host ecology, biology, and/or vector preferences may drive differential parasite burdens in this host community. Since most of these birds were caught in the same area it is likely that their exposure to the infected vector is similar but the variation in their ecology, such as microhabitat choice, variation in feeding preferences of the dipteran vector, and/or variation in unprofiled aspects of their immune response to infection could underlie these differences. Other studies of Galápagos birds similarly demonstrate that species vary in their susceptibility and their response to introduced parasites and pathogens (Heimpel et al., 2017; Knutie et al., 2016; McNew et al., 2022). However, in many cases the mechanisms underlying this variation remains to be explored.

Finally, we investigated potential health impacts of infection on host health. We found no relationship between microfilarial infection and white blood cell counts, H/L ratios, or body condition. Other species of microfilarial nematodes are known to have immunomodulatory mechanisms which help the parasite hide from the host immune system (Garrido-Bautista et al., 2023). It is also possible that this parasite is a recent introduction, and these bird species have not yet evolved an effective immune response to the parasite, leading to the lack of immune reaction as observed in infected birds. On the other hand, it is also possible that this microfilaria has been present on the island with these bird populations for a long time, leading to coevolution where the birds tolerate infection without presenting disease symptoms. Further studies could investigate whether variation in parasite burden and/or timing of infection has any effect on immune response or host condition.

### 4.3 Conclusion

The Galápagos Islands are home to many endemic and iconic species that are experiencing significant ecological changes, including the emergence of disease. Introduced parasites are one of the most significant threats to Galápagos birds. While attention has focused on the introduced parasite *P. downsi*, continued surveillance is needed to anticipate and respond to other emerging parasites and pathogens. Even parasites with minor effects on host fitness could have significant impacts on struggling populations, such as the little vermilion flycatcher (*Pyrocephalus nanus*) on Santa Cruz Island and mangrove finch (*Camarhynchus heliobates*) on Isabela Island. We found a previously undescribed species of *Eufilaria* nematode infecting several species of passerines in the lowland habitat of Santa Cruz. Although we did not detect any correlation with infection and any measurable health parameters, we did find that prevalence varied among host species. More research is needed to determine the driving factors behind the variation in prevalence among species, as well as to better understand the origin, transmission dynamics, and fitness consequences of these parasites.

## CRediT authorship contribution statement

**Diana Carolina Loyola:** Writing – original draft, Methodology, Investigation, Data curation, Conceptualization. **Allyson Placko:** Writing – review & editing, Writing – original draft, Visualization, Validation, Project administration, Methodology, Investigation, Formal analysis, Data curation. **Birgit Fessl:** Writing – review & editing, Supervision, Project administration, Investigation. **Sabrina M. McNew:** Writing – review & editing, Supervision, Project administration, Methodology, Investigation, Funding acquisition, Data curation, Conceptualization.

## Conflict of interest

The authors declare no conflict of interest.

## Data Availability

All sequences have been deposited in GenBank and are available under accession numbers PV595630 – PV595659. Code and data used in the analysis available via Zenodo https://doi.org/10.5281/zenodo.15793823.
