# Supplementary material for: Novel microfilariae detected in Galápagos passerines
Source: Int J Parasitol Parasites Wildl. 2025 Jul 12;28:101115. doi: 10.1016/j.ijppaw.2025.101115 (PMC12302254; doi:10.1016/j.ijppaw.2025.101115)
Supplement: Multimedia component 1 [file mmc1.docx]

Table S1. Details for all microfilaria samples sequenced in this study.

| Accession | Sequence ID | Host ID | Host Species | Haplotype |
| --- | --- | --- | --- | --- |
| PV595630 | SM2635_MF1 | SM2635 | *Platyspiza crassirostris* | MF1 |
| PV595631 | SMC024_MF1 | SMC024 | *Mimus parvulus* | MF1 |
| PV595632 | SM2474_MF2 | SM2474 | *Platyspiza crassirostris* | MF2 |
| PV595633 | SMC076_MF1 | SMC076 | *Mimus parvulus* | MF1 |
| PV595634 | SMC093_MF1 | SMC093 | *Geospiza fortis* | MF1 |
| PV595635 | SM2160_MF1 | SM2160 | *Geospiza fortis* | MF1 |
| PV595636 | SMA2156_MF2 | SMA2156 | *Geospiza fuliginosa* | MF2 |
| PV595637 | SMA1521_MF1 | SMA1521 | *Camarhynchus parvulus* | MF1 |
| PV595638 | JP4878_MF1 | JP4878 | *Platyspiza crassirostris* | MF1 |
| PV595639 | SMC038_MF2 | SMC038 | *Mimus parvulus* | MF2 |
| PV595640 | SM2435_MF1 | SM2435 | *Platyspiza crassirostris* | MF1 |
| PV595641 | SMC096_MF1 | SMC096 | *Mimus parvulus* | MF1 |
| PV595642 | SMA2039_MF2 | SMA2039 | *Camarhynchus parvulus* | MF2 |
| PV595643 | SM2165_MF1 | SM2165 | *Geospiza fortis* | MF1 |
| PV595644 | SMA1848_MF1 | SMA1848 | *Geospiza fuliginosa* | MF1 |
| PV595645 | SM1795_MF2 | SM1795 | *Geospiza scandens* | MF2 |
| PV595646 | SM1954_MF1 | SM1954 | *Platyspiza crassirostris* | MF1 |
| PV595647 | SM2108_MF1 | SM2108 | *Platyspiza crassirostris* | MF1 |
| PV595648 | SM2647_MF2 | SM2647 | *Platyspiza crassirostris* | MF2 |
| PV595649 | SMC026_MF2 | SMC026 | *Mimus parvulus* | MF2 |
| PV595650 | SMA2136_MF1 | SMA2136 | *Camarhynchus parvulus* | MF1 |
| PV595651 | SMC071_MF1 | SMC071 | *Geospiza fortis* | MF1 |
| PV595652 | SMA2223_MF1 | SMA2223 | *Geospiza fuliginosa* | MF1 |
| PV595653 | SM2120_MF2 | SM2120 | *Geospiza scandens* | MF2 |
| PV595654 | SM1876_MF1 | SM1876 | *Geospiza scandens* | MF1 |
| PV595655 | SM2101_MF2 | SM2101 | *Platyspiza crassirostris* | MF2 |
| PV595656 | SM2750_MF2 | SM2750 | *Platyspiza crassirostris* | MF2 |
| PV595657 | SM1766_MF1 | SM1766 | *Platyspiza crassirostris* | MF1 |
| PV595658 | SM2294_MF1 | SM2294 | *Geospiza fortis* | MF1 |
| PV595659 | SM1923_MF1 | SM1923 | *Geospiza scandens* | MF1 |
